# Titania Nanotube Architectures Synthesized on 3D-Printed Ti-6Al-4V Implant and Assessing Vancomycin Release Protocols

**DOI:** 10.3390/ma14216576

**Published:** 2021-11-01

**Authors:** H-thaichnok Chunate, Jirapon Khamwannah, Abdul Azeez Abdu Aliyu, Saran Tantavisut, Chedtha Puncreobutr, Atchara Khamkongkaeo, Chiraporn Tongyam, Krittima Tumkhanon, Thanawat Phetrattanarangsi, Theerapat Chanamuangkon, Torlarp Sitthiwanit, Dechawut Decha-umphai, Pharanroj Pongjirawish, Boonrat Lohwongwatana

**Affiliations:** 1 M3D Laboratory, Advanced Materials Analysis Research Unit, Department of Metallurgical Engineering, Faculty of Engineering, Chulalongkorn University, Bangkok 10330, Thailand; hthaichnok721@gmail.com (H.-t.C.); Jkhamwan@gmail.com (J.K.); garoabdul@gmail.com (A.A.A.A.); chedtha@gmail.com (C.P.); atchara.k@gmail.com (A.K.); ma_uksa@hotmail.com (C.T.); t.krittima@gmail.com (K.T.); thanawat@meticuly.com (T.P.); torlarpsitthiwanit@gmail.com (T.S.); dechawut@meticuly.com (D.D.-u.); 2 Biomedical Engineering Research Center, Chulalongkorn University, Bangkok 10330, Thailand; 3 Hip Fracture Research Unit, Department of Orthopaedic, Faculty of Medicine, Chulalongkorn University, Bangkok 10330, Thailand; super_petch@yahoo.com; 4 Biomechanics Research Center, Meticuly Co. Ltd., Pathumwan, Wang Mai District, Bangkok 10330, Thailand; pharanroj@meticuly.com; 5 Biomaterial Testing Center, Faculty of Dentistry, Chulalongkorn University, Bangkok 10330, Thailand; teera.pat.n61@gmail.com

**Keywords:** 3D printing, Ti-6Al-4V, titania nanotubes, vancomycin, electrostatic interaction

## Abstract

The aim of this study is to synthesize Titania nanotubes (TNTs) on the 3D-printed Ti-6Al-4V surface and investigate the loading of antibacterial vancomycin drug dose of 200 ppm for local drug treatment application for 24 h. The antibacterial drug release from synthesized nanotubes evaluated via the chemical surface measurement and the linear fitting of Korsmeyer–Peppas model was also assessed. The TNTs were synthesized on the Ti-6Al-4V surface through the anodization process at different anodization time. The TNTs morphology was characterized using field emission scanning electron microscope (FESEM). The wettability and the chemical composition of the Ti-6Al-4V surface and the TNTs were assessed using the contact angle meter, Fourier transform infrared spectrophotometer (FTIR) and the X-ray photoelectron spectroscopy (XPS). The vancomycin of 200 ppm release behavior under controlled atmosphere was measured by the high-performance liquid chromatography (HPLC) and hence, the position for retention time at 2.5 min was ascertained. The FESEM analysis confirmed the formation of nanostructured TNTs with vertically oriented, closely packed, smooth and unperforated walls. The maximum cumulative vancomycin release of 34.7% (69.5 ppm) was recorded at 24 h. The wetting angle of both Ti-6Al-4V implant and the TNTs were found below 90 degrees. This confirmed their excellent wettability.

## 1. Introduction

The cases related to bone fracture operations are progressively rising annually. This is due to an annual increase in aging population and traffic accidents. Thus, there is increasing demand for bone fixation implants such as plate or intramedullary nail. The most common materials used to fabricate orthopaedic implants include stainless steel, cobalt-chromium, titanium and its alloys [1,2,3].

Peri-implant infection is one of the most serious complication after surgery. Aside strict sterilization procedures, the rate of bacterial infection is reported at 1 to 4% in previous literature [4]. The economics of global burden for the treatment of orthopedic infection and the additional payment from implant surgeries consumed more than $1.6 billion in 2020 [5]. Implant infection may results to multiple re-operations, revision surgeries, limb loss or death. The current standard treatment used in most hospitals is time consuming, unpredictable and not enough for reduction of an implant infection. The totally controlled infection rate for intravenous (IV) and oral antibiotics after internal fixation surgery was reported at 83.6% [6]. The systemic drug delivery (SDD) deliver less than 1% of the administered drug to the infected site [7]. To increase the antibiotics concentration at infection site, the local antibiotics delivery system such as antibiotic loading bone cement is applied. The major concerns with the antibacterial drug loaded Polymethyl methacrylate (PMMA) cement beads are releasing performance lower than minimum inhibitory concentration (MIC) treatment and cytotoxicity [8,9].

Another route for drug delivery and considered most promising is the localized drug delivery (LDD) system. This system deliver antibiotic to the specific area. In LDD, drugs like vancomycin, gentamicin, tobramycin and cephalosporins are incorporated in the implant and administered at a local infection site in a controlled manner, thereby decreasing the infection rate of the repaired fracture. Antibacterial drug loading into the porous structure of titania nanotubes (TNTs) allows sufficient drug loading and subsequent release on the surgical local site. This approach remained a key strategy to minimize or mitigate the implant infection problem [10,11]. Recently, Kunrath et al. [12] and Kunrath et al. [13] presented the state-of-the-art reviews and critically analyzed the use of TiO_2_ nanotube coated on the various biomedical implant surfaces. It is confirmed that TNTS are biocompatible, an excellent site for tissue ingrowth into the porous structure and allows strong cells adhesion and proliferation. TNTs also serve as a promising route for incorporating specific drug into the nanotubes and regulate the release of such drug to the infected site. In an attempt to control the release of vancomycin loaded TNTs into the diseased tissues, electrophoretic technique was proposed [14]. This technique was proven viable for high capacity vancomycin loading and releasing control. Despite that, some sporadic reports on anodization of Ti-6Al-4V for LDD and release control could be found; there still need for more studies that will focus on various techniques for synthesizing TNTs, especially on the 3D-printed implants and analyzing the drug loading capacity of such TNTs. Hence, the motivation for this study.

In this study, the anodization technique was used to synthesize titania nanotubes arrays on the 3D-printed Ti-6Al-4V surface. The aim is to load the antibacterial vancomycin into the synthesized titania nanotubes and analyze its release performance for a period of 24 h. This is expected to serve as a local drug treatment to the infection at the surgical site. Various characterization tools such as field emission scanning electron microscopy (FESEM), contact angle meter, Fourier transform infrared (FTIR), and atomic force microscope (AFM) were employed to analyze the morphology, wettability behavior functional group and the topography of the synthesized TNTs surface, respectively.

## 2. Materials and Methods

### 2.1. Materials and Anodization

**Figure 1:**
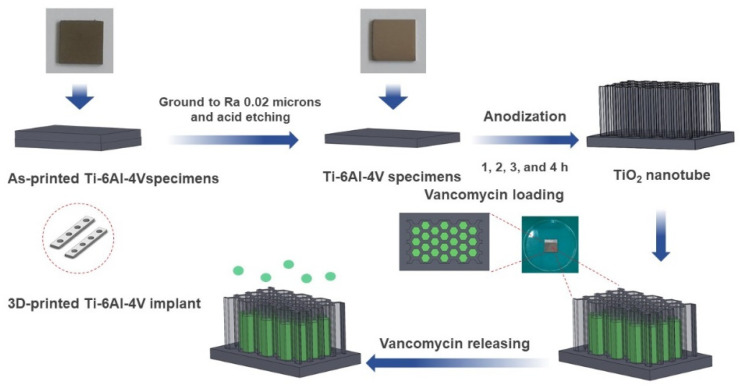
A schematic diagram depicting a stage-by-stage the vancomycin release protocol from the fabricated titania nanotubes surface on the 3D-printed Ti-6Al-4V implant material. The as-printed specimen was ground and chemically etched to a suitable roughness surface. The titanium oxide nanotubes (TNTs) were synthesized at different at anodization duration.

**Figure 2:**
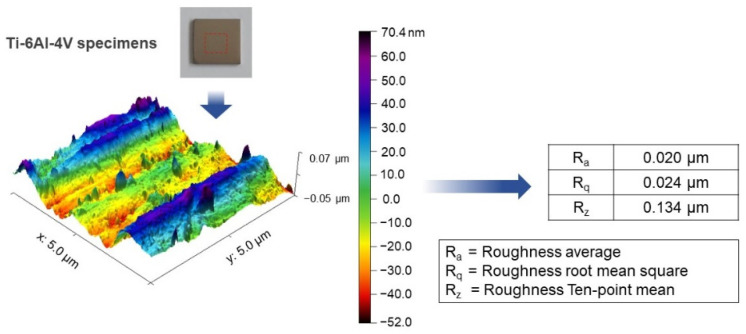
Atomic force micrograph showing the 3D surface topography of the 3D-printed Ti-6Al-4V specimen prior to the TNTs fabrication through the anodization process. Various measured roughness values were presented.

SolidWorks 2020 software was employed to design and model the Ti-6Al-4V plate implant (25 mm × 25 mm × 2 mm). The implant model is converted to STL file and fabricated using D50 micro size Ti-6Al-4V powder by SLM (Model: Mlab cusing 100R, Concept Laser GmbH, Lichtenfels, Germany) technique, Meticuly Company, Bangkok, Thailand. The Ti-6Al-4V plate implant is used as the anode and a commercial platinum plate (Umicore, Pforzheim, Germany) with size 12 mm x 30 mm was employed as the cathode, during the anodization process. Under controlled atmosphere in an electric furnace (Nabertherm N7/H, Lilienthal, Germany), the Ti-6Al-4V plate specimens were heat treated at 950 °C for 2 h. Prior to anodization, the surface asperity was reduced by conventional grinding with 80 to 2000 grit paper and sonication within the acid solution, deionized water, and ethanol. To achieve nanostructured surface and orderly arranged TNTs arrays with a very high aspect ratio, fluoride containing polyhydric alcohols, specifically Ethylene glycol was employed as the electrolyte. Another reason for selecting ethylene glycol-based electrolyte, its characteristics in producing a biocompatible and bioactive surface [15]. The ground specimens were immersed into the prepared ethylene glycol-based electrolyte containing a mixture solution of Ethylene glycol 98 wt.% (Qrec, Auckland, New Zealand), Ammonium fluoride (0.5 wt.%) (Qrec, Auckland, New Zealand) and 1.5 wt.% deionized water for the duration of 1, 2, 3 and 4 h. The electrolyte solution was continuously stirred with a magnetic bar at 100 rpm. Figure 1 shows the schematic illustration of the overall methodology involved in this study. Figure 2 presents the three-dimensional surface topography of the 3D-printed Ti-6Al-4V specimens before anodization process. The printed Ti-6Al-4V surface has heterogeneous interface of the native oxide layer due to oxidation reaction between of alloy samples and the atmosphere.

### 2.2. Titania Nanotubes Characterization

The morphology and nanostructure of the anodized titania nanotube film was investigated using field emission scanning electron microscope (FESEM, FEI Quanta FEG 250, Thermo Fisher Scientific, Hillsboro, OR, USA) under 15 kV. The wettability characteristic and the interactions between the ions of the titania film were evaluated using contact angle meter and Fourier transform infrared (FTIR, Thermo Fisher Scientific, Hillsboro, OR, USA), respectively. The Atomic Force Microscope (AFM, Veeco, Dimension 3100, Plainview, NY, USA) was used to observe the nanostructure and surface roughness of the 3D-printed Ti-6Al-4V plate. The elements presence on the coated surface was investigated using X-ray photoelectron spectroscopy (XPS, Bara Scientific Co., Ltd., series Axis Ultra, Bangkok, Thailand). SPSS 22.0 software (IBM, Armonk, NY, USA) was used to analyzed the pores diameter and the length of the TNTs.

### 2.3. Drug Loading

As a drug model, vancomycin was selected, because it is the most powerful in treating serious bacterial infections. Prior to loading of the vancomycin, the deposited nanotubes surface was cleaned with 0.1 m HCl, and ethanol (Qrec, Auckland, New Zealand) to reduce the scale oxide. The vancomycin solution was prepared using 200 ppm concentration as recommended by Fleischman et al. [16]. The 0.5 g of vancomycin powder (Vancin-S, Siam Pharmaceutical, Bangkok, Thailand) was dissolved in 10 mL of sterile water (SWI) for the injection (PIC, Thailand). It was then diluted until the drug concentration of 200 ppm solution is achieved. Briefly, 2000 mg of vancomycin was dissolved in 10 mL phosphate buffer solution (PBS) resulting in 200 mg/mL. Then, the drug solution of 10 mL was directly pipetted onto the nanotubes surface for 20 min. Delicate task wipers (Kimberly-Clark, Irving, TX, USA) were used to reduce the extra drug on the titania nanotubes surface. After the vancomycin loading, the specimens were sealed and covered with aluminum foil (Diamond, IL, USA) for light protection.

### 2.4. Drug Releasing Analysis

In this stage, the vancomycin loaded specimens were immersed in 10 mL of phosphate buffer solution (PBS) under controlled rotational speed and incubation temperature of 50 rpm and 37 °C, respectively. The drug solution was filtrated with 0.45 μm nylon filter (Shimadzu, Kyoto, Japan) before injection to chromatography analysis. To determine the release behavior, three samples of incubated solution containing 25 µL was prepared and injected at once, in a specific time. The initial vancomycin release was taken at 1 h. Subsequent vancomycin release was taken at various time intervals. The percentage vancomycin release was determined by taking the ratio of the amount of the vancomycin released into PBS to that of the total vancomycin loaded in the TNTs and multiplied by 100. The real concentration of vancomycin was calculated from the concentration of vancomycin released in ppm multiplied by dilution factor (1.4). The vancomycin drug release versus the calibration curve for vancomycin solution of 0.5, 1, 2.5, 5, 10, 25, 50, and 100 ppm was plotted.

The High-Performance Liquid Chromatography (HPLC) (LC-20 Series, Shimadzu, Japan) was used for the qualitative analysis of the vancomycin released in the water-based salt solution. The HPLC machine comprises of a pump (LC-20AD), auto injector (SIL-20AC HT) and UV detector (SPD-M20A) at 240 nm. The mobile phase was consists of acetonitrile (HPLC grade, Sigma-Aldrich, USA, ultra-pure water (20:80, *v*/*v*), which adjusted the pH value by 0.1% *v*/*v* formic acid (Qrec, Auckland, New Zealand) with a flow rate of 1 mL/min. The column condition was C18 column (150 × 4.6 mm, 5 μm, Shim pack GIST, Shimadzu, Kyoto, Japan) and the oven temperature of 25 °C.

## 3. Results

### 3.1. Morphology of TiO_2_ Nanotube Layer

**Figure 3:**
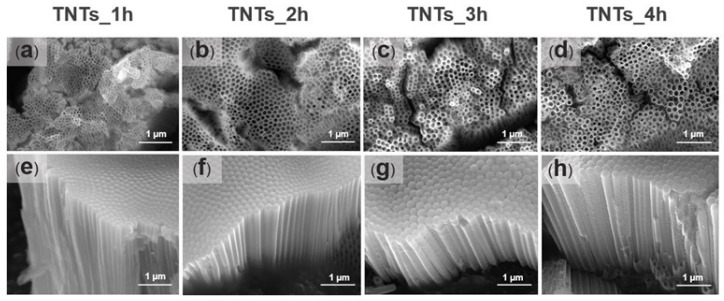
The FESEM micrographs showing the top and side morphological views of the titanium oxide nanotubes fabricated at 1 h (**a**,**e**), 2 h (**b**,**f**), 3 h (**c**,**g**) and 4 h (**d**,**h**). The morphology images depict the TNTs orientation and the size of the pores. The pores diameter of the nanotube increased with increase in the anodization time while the length decreased.

Prior to surface morphology examination, the anodized titania implants were etched with 0.1 M HCl to reduce the scale oxide, which covers the surface of the deposited titania nanotubes array. Figure 3a–h shows the FESEM micrographs of the TNTs anodized at 1, 2, 3, and 4 h. The TNTs were vertically oriented on the Ti-6Al-4V substrate material, closely packed with smooth and unperforated walls in all the anodized titania implants. Numerous nanostructures could be noticed in all the anodized titania implants. The nanotubes pore diameter and the length presented in Table 1 was measured using Image J software version Java 8 (National Institutes of Health, Bethesda, MD, USA). The pores diameter increased from 53 nm to 108 nm when the anodization time increased to 2 h while the nanotube length decreased from 1976 nm to 1378 nm. On the other hand, the diameter decreased from 108 nm to 93 nm, and the nanotubes length increased 1934 nm to 2629 nm when the time was further increased to 3 h. The lowest pore diameter was observed at anodization time of 1 h (53 nm) while the highest at 4 h (114 nm). Thus, some fluctuations could be observed in the nanotubes diameter and the length, which might relate to the uneven titania surface formed due to the native oxide layer in the Ti-6Al-4V substrate. The result obtained is in agreement with that of Hamlekhan et al. [17], which fabricated an anti-aging titania nanotubes on the titanium surface.

To assess the effect of each factor (anodization time) on the variation in the pores diameter and the length of the TNTs, statistical analysis was conducted using IBM SPSS 22.0 software. Table 1, Table 2 and Table 3 shows the One-way ANOVA results for the pores diameter and the length of the TNTs, respectively. It is confirmed that the independent effect of all the anodization times have an F-value much larger than 1 and a *p*-value considerably lower than 0.05. This indicated that the effect of each anodization time on the pores diameter and the TNTs length is statistically significant.

### 3.2. Hydrophilicity of TNTs Surface

**Figure 4:**
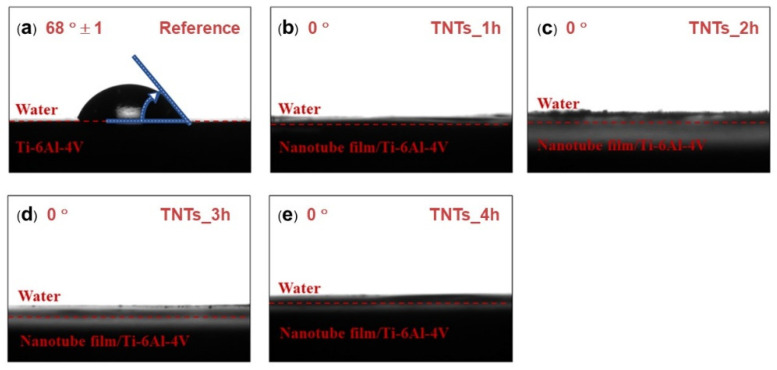
Water contact angle of (**a**) as-printed Ti-6Al-4V, (**b**) TNTs_1h, (**c**) TNTs_2h, (**d**) TNTs_3h and (**e**) TNTs_4h. The wetting angle of “0” degrees were found in all nanotube surfaces, suggesting the high hydrophilicity of the fabricated TNTs surface.

Contact angle measurement was employed to study the wettability of the TNTs nanoporous surface. The contact angle of the 3D-printed Ti-6Al-4V and the TNTs synthesized at different anodization time is depicted in Figure 4. The wetting angle of both the 3D-printed Ti-6Al-4V and the TNTs was found below 90 degrees (θ < 90°). The 3D-printed Ti-6Al-4V, which is the reference surface have the largest contact water angle (68°) and the anodization area as shown in Figure 4a. All the TNTs have the lowest contact angle of 0° which confirmed their superhydrophilic characteristics, this might be attributed to the highly rough and nanoporous surface presence in the TNTs surface. Thus, the synthesized TNTs are expected to have excellent tissues adhesion and cells proliferation [18].

### 3.3. Functional Group of Nanotubes Film

**Figure 5:**
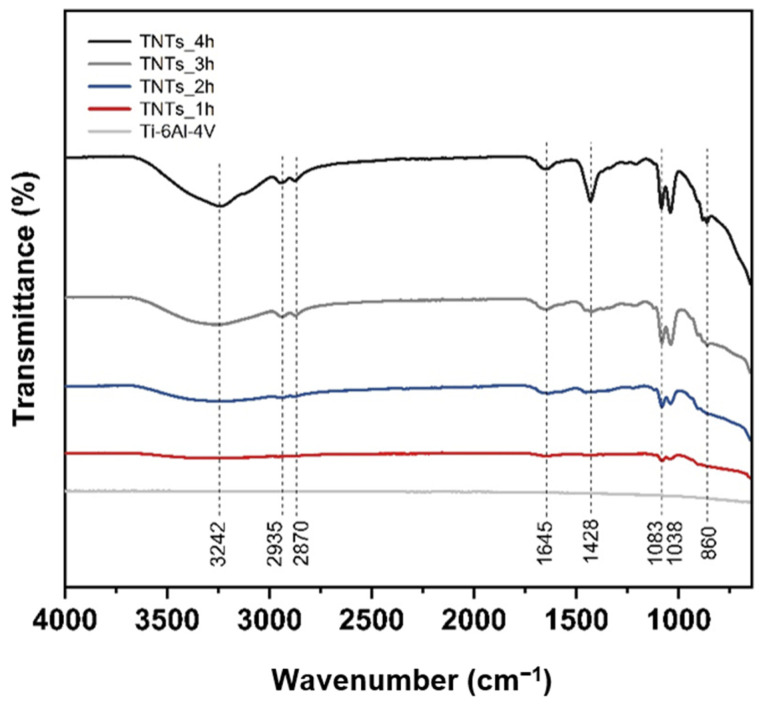
The ATR-FTIR spectra of the 3D-printed Ti-6Al-4V and the synthesized TNTs_1h, TNTs_2h, TNTs_3h and TNTs_4h at a different anodization time and scanning range of 4000–600 cm^−1^, containing various functional groups, which signifies the chemical properties of each specimen.

The ATR-FTIR spectra of the 3D-printed Ti-6Al-4V and TNTs at a different anodization time in a scanning range of 4000–600 cm^−1^ is depicted in Figure 5. Various functional groups indicating the chemical properties of each specimen could be observed. The broadband position at 3242 cm^−1^ is the stretching vibration of the hydroxyl group, which is incorporated with the titania nanotubes interface. This is due to polyhydric alcohols (ethylene glycol) used during the anodization, which contain OH functional group [19]. The slight doublet bands at 2935 and 2870 cm^−1^ are CH_2_ stretching vibration. The weaken band located at 1645 cm^−1^ presented the bending vibration mode of hydroxyl Ti–OH [15,20]. The band at 1428 cm^−1^ corresponding to CH_2_ stretching vibration and strongly peak of TNTs_4h. The closely double bands position at 1083 and 1038 cm^−1^ are C–O stretching vibration. The widely broad band area at 860 cm^−1^ is Ti–O and Ti–O–Ti stretching vibration [21,22,23]. The peak of the hydrophilic group with hydrogen bonding increases the percentage of the transmittance when high duration time is applied.

### 3.4. Oxide Species Analysis

**Figure 6:**
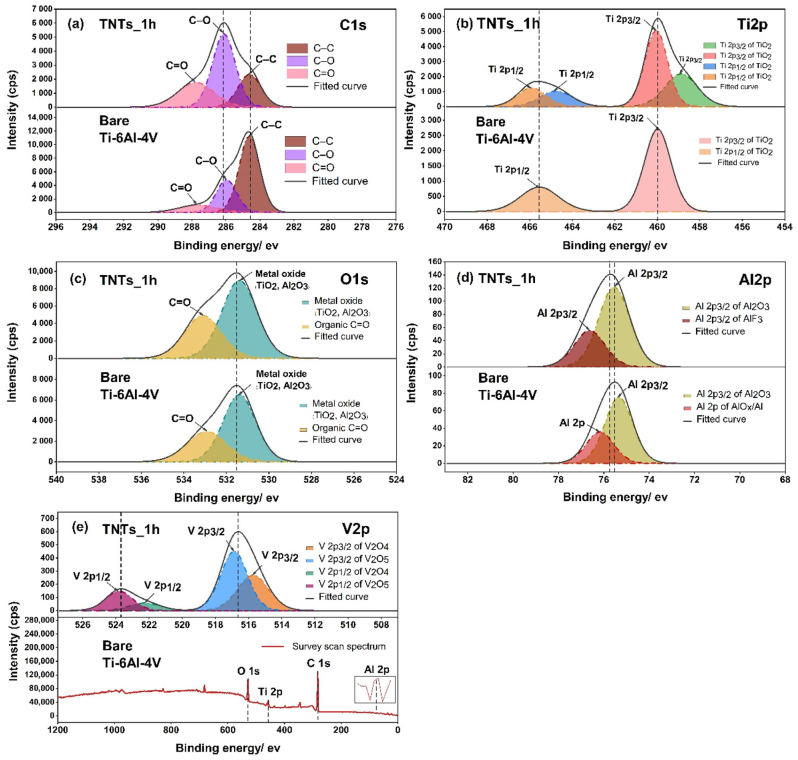
The XPS spectrums presenting the chemical composition of the oxide species of pre-post anodization process on the printed Ti-6Al-4V surface and the nanotubes of (**a**) C1s, (**b**) Ti2p, (**c**) O1s, (**d**) Al2p and (**e**) V2p, and the survey scan spectra for V2p.

To analyze the surface chemistry of the 3D-printed Ti-6Al-4V and the TNTs, XPS spectra was employed as presented in Figure 6. The C1s peak, which has three components including carbon bonded to carbon and oxygen located at 284.6 eV (C–C), 286.0 eV (C–O) and 287.8 eV (C=O) were noticed. The presence of carbon on the TNTs surface may attributed to the carbide cutting tool used for cutting the specimens. The Ti2p peak of the anodized TNTs_1h film shown in Figure 6b consists duplet of Ti2p_3/2_ and Ti2p_1/2_ peaks at 458.8, 460.0, 464.7, 465.9 eV respectively. The binding energies of 458.8 and 464.7 eV reported as a Ti^4+^ species of oxidation state [24,25], while the position of 460.0 and 465.9 eV represents Ti^3+^ species of two oxides (TiO_2_ and Ti_2_O_3_) [26]. The 3D-printed Ti-6Al-4V surface shows the binding energies of 459.9 and 465.5 eV, which illustrated chemical species as Ti^3+^ of different oxide component TiO_2_ and Ti_2_O_3_. In Figure 6c, the O1s regions of the central peak at 531.3 and 531.4 eV of the nanoporous TNTs_1h and 3D-printed Ti-6A-4V surface respectively, indicated the formation of the metal oxide (TiO_2_, Al_2_O_3_) and organic compound (C=O). This resulted in the growth of titania nanotubes to Ti–O bonds and absorption of a hydroxyl group (–OH) from polarity solvent, aqueous molecules and the atmosphere [25]. Moreover, the Al_2_O_3_ arises from the oxidation reaction of Al and O during the electrochemical process [27]. On the modified and unmodified surfaces, the organic carbon compound (C=O) covered the oxide surface layer which is located at 533.4 and 532.9 eV. For a vanadium oxidation state in the high resolution O1s spectrum, there is no chemical species observed. This may be due to very low signal in the combined peak of Ti and Al elements. Hence, the vanadium oxide in the narrow scan of V2p should be analyzed in the later stage. The deconvolution of the high Al2p spectrum in Figure 6d displayed the split two bands of Al2p at 75.5 and 75.3 eV for TNTs and the 3D-printed Ti-6Al-4V surface, represented by Al2p_3/2_ of Al_2_O_3_. Additionally, the bands of Al2p at 76.6 and 76.1 eV contained AlF_3_ and Al2p of AlO_x_/Al (Ceramic/Metal) based on NIST X-ray photoelectron database with the binding energies tolerance of ± 0.2 [28]. Figure 6e shows the elemental analysis of V2p which involved V2p_1/2_ and V2p_3/2_. The high resolution of V2p of TNTs_1h represented a double phase of the vanadium oxide with first oxide species located at 515.7 and 522.2 eV, consisting of V_2_O_4_. The second chemical component of oxide film at 516.8 and 523.7 eV contained V_2_O_5_.

### 3.5. Antibacterial Drug Release Behavior

**Figure 7:**
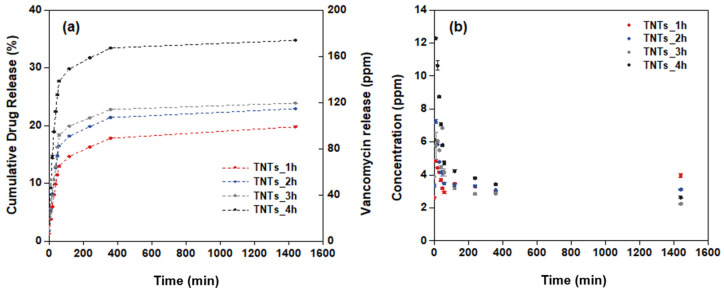
Drug release profiles of (**a**) the percentage of cumulative drug release and (**b**) the concentration of drug release at different anodization time of the titanium oxide nanotubes.

Under environmental control, the antibacterial vancomycin drug was released into the titania nanotubes surface. The amount of the vancomycin solution, which is light-sensitive was measured using chromatography technique by the HPLC machine system. The mobile phase shows the retention time of the vancomycin as 2.5 min. The quantitative analysis of the antibacterial drug released from the titania nanotubes surface to the stimulated media containing the PBS solution was calculated as the percentage cumulative of the vancomycin release for 24 h. The calculated drug release profile is elaborated in Figure 7a. The drug releasing behavior involved two phases; burst and constant releasing. In the first stage, the drug is released from the nanopores of the TNTs gradually until the highest vancomycin concentration is achieved at 24 h. The initial concentration of the vancomycin released are 2.6, 3.4, 4.1 and 6.1 ppm for the TNTs_1h, TNTs_2h, TNTs_3h and TNTs_4h respectively. In all the TNTs, the vancomycin increases with increase in the release time. Out of the total drug loaded, the cumulative vancomycin release at 24 h is 19.8% (39.6 ppm), 22.9% (45.8 ppm), 23.9% (47.8 ppm), and 34.7% (69.5 ppm) of the TNTs_1h, TNTs_2h, TNTs_3h and TNTs_4h respectively. This confirmed the highest drug release by TNTs_4h and the capability of the synthesized nanotubes on the Ti-6Al-4V surface to enhanced the release of vancomycin drug in the injured area. Figure 7b shows the real concentration while the antibacterial drug releasing is observed. The decreasingly behavior of the vancomycin concentration was observed until 4 h, then it continues constantly up to 24 h.

## 4. Discussion

Several anodization process parameters such as chemical composition of the electrolyte, voltage, atmospheric temperature, crystal structure, and chemical component of Ti-6Al-4V affect the physical properties and morphology of the titania nanotube surface layer. In this study, nanotubular titania oxide films were fabricated with varying anodization duration. The numerical data (Table 1) obtained from the nanostructural observation using FESEM micrographs depicts that, increasing anodization time to 2 h, resulted in bigger inner pore diameter while the length of the synthesized titania nanotubes decreases. However, the diameter of the nanotubes decreased when the time increased to 3 h, while the nanotubes length increased. At anodization time of 4 h, the nanotubes diameter further increased with slight decrease in the length. Thus, compared to TNTs anodized at 1, 2 and 3 h, those anodized at 4 h have more potential of higher loading capacity of the antibacterial drug for the treatment of the infection site [29].

The 3D-printed Ti-6Al-4V contained a native oxide layer which is formed by α and β phases [30]. These phases in the 3D-printed Ti-6Al-4V substrate affect the surface topography of this material (Figure 2). During anodization process, the β phase is easily etched by the electrolyte charges than the local oxide of α phase. Therefore, the titania nanotubes are distinctly separated by these two-phase sites. The highly ordered nanoporous architecture is originated from the α phase while the disordered nanostructure was related to the β phase. Similar finding was also reported by Wang et al. [31,32].

The titania nanotubes synthesized on the Ti-6Al-4V surface differs in inner pore diameter and the length due to oxidation and dissolution mechanism of metal oxide on the alloy interface during the anodization process [33]. When the voltage is applied for the oxide layer synthesis, its surface interact with the oxygen ions (O^2−^) in the electrolyte [33]. The β phase show higher activities than the α phase at the voltage of 70–103 V for the alloy surface dissolution with an oxygen atom [34]. Numerous nanotube structures were observed on the substrate surface after anodization was carried out at different duration (Figure 3 and Table 1). Varying the anodization time between 2–4 h resulted in the formation of hexagonal nanopores structure and vertically oriented titania nanotubes. The anodization time affect the pores shape and size of the synthesized TNTs. The pores diameter are smaller, highly packed and more densely packed TNTs were observed when anodized at 1 h (Figure 3a). The hexagonal packing of the TNTs appeared more clearly with larger pores diameter when the time increased to 2 h (Figure 3b). The pores shape and size appeared much more clear when anodized at 3 and 4 h (Figure 3c,d, repectively).

Additionally, by maintaining the electrochemical parameters at 60 V while varying the anodization time, the growth of metallic nanotubes encourages the F^-^ ions formation from the aqueous-based electrolyte and dissolved TiO_2_ compact layer between anode and the solution interface [35]. In the chemical dissolution stage, the formation of titania nanotubes resulted in the fluoride ion in the form of soluble hexafluoro titanium complex [TiF_6_]^2−^. This [TiF_6_]^2−^ is an important etchant, which normally dissolved^-^ in the electrolyte and hence, not detected during the XPS analysis [36].

The results in Figure 4 showed a good wettability of the TNTs synthesized on the Ti-6Al-4V implant surface. This is due to the nanoporous nature of the titania film whereby the water infuses into the porous structure simply [37]. This decreased the contact angle of the 3D-printed Ti-6Al-4V from 68° ± 1° to 0°. The hydroxyl and the hydrophilic groups formed on the anodized specimen surface is attributed to the amorphous nature of titania nanotubes as shown in Figure 5. Surface treatment (annealing) after anodization might reduce the amorphous structures, including high density of the hydrophilic groups and the polarity bonding of O–Ti–O [31,33]. The pore structure of TiO_2_ nanotubes and the functional group on the nano surface making the anodized film layer more wettable corresponding to the Wenzel’s model. Moreover, the higher pore size diameter causes a more capillary force, surface energy, surface area and site for aqueous infiltration [38,39,40,41].

Numerous oxide species such as Al2p and V2p based were observed on the anodized 3D-printed Ti-6Al-4V surface (Figure 6). This is attributed to chemical dissolution between electrolyte and the 3D-printed Ti-6Al-4V interface. Thus, Ti^4+^ and F^-^ are transferred to the specimen surface thereby etching the oxide surface during the titania nanotubes formation [31,32,33,42]. Other compounds resulted from the anodization of the 3D-printed Ti-6Al-4V includes AlF_3_, V_2_O_4_ and V_2_O_5_. These compounds occurred under controlled electrolyte and voltage potential.

**Figure 8:**
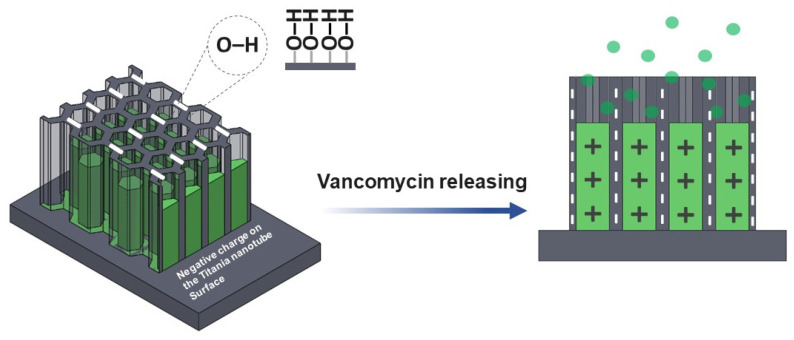
The model of vancomycin release is proposed. The hydroxyl group (OH) and other functional hydrophilic group from the ATR-FTIR characterization donated by negatively charge ions is attributed to the nanotube surface after anodization.

The chemical properties of the nanostructured interface contained negatively charged ions which are suitable for positively charged vancomycin absorption via electrostatic interactions [29,43]. Varying the anodization time affects the drug release behavior (Figure 7a) higher than the vancomycin MICs for 2 ppm (2 μg/ml) [44,45]. This is because the water-soluble drug that was loaded in the TNTs will interact with the functionalize interface (Figure 8). The longer nanotube has high surface area for this interaction; therefore, the drug moving out from nanostructure slower than shorter nanotube.

**Figure 9:**
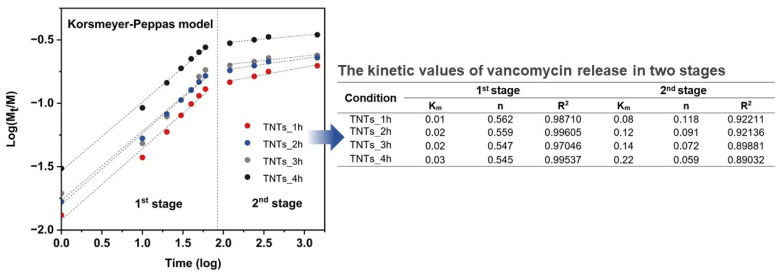
The measured values of vancomycin release was fitted to Korsmeyer-Peppas model which describes the behavior in two main stages. The tabulated fitting the kinetic values of K and n from Korsmeyer-Peppas model, which described the kinetics of drug release in two stages. The 1st stage is the anomalous diffusion or Non-Fickian diffusion in the burst stage in which the n values are in the range of 0.54–0.56 while the 2nd stage shows the n values in the range of 0.05–0.12 suggesting Quasi-Fickian diffusion behavior.

The drug releasing mechanism was evaluated by using Korsmeyer-Peppas model [46] and linear fitting for finding K_m_ (Kinetic constant) and n (Release exponent) values. Figure 9 presented K_m_ and n value fitted in the following equations:

F = (M_t_/M) = K_m_·t^n^ (1)

Log (M_t_/M) = Log K_m_·t^n^ (2)

where F is the fraction of drug released at a time, M_t_ is the amount of drug release, M is the total amount of drug in dosage form, K_m_ is kinetic constant, n is diffusion or release exponent, and t is the time for drug releasing (min). The n value involved the morphology of the drug reservoir and can interpret the drug release mechanism according to the various diffusion types. For instance, when n = 0.5 the release is Fickian diffusion, while 0.5 < n < 1 is the anomalous diffusion (non-fickian). In some cases, if n = 1 and n > 1, the diffusion mechanism is case-II transport and super case-II transport, respectively.

Investigation of K_m_ and n values was appraised by the linear logarithm from Korsmeyer-Peppas equation. Figure 9 revealed the vancomycin release profile, which is divided into two stages for all the anodized specimens and presented K_m_ and n values that indicated the mechanism of the antibacterial vancomycin transport as the anomalous diffusion or Non-Fickian diffusion in the burst stage in which the n values are in the range of 0.54–0.56. The second stage shows the n values in the range of 0.05–0.12 suggesting Quasi-Fickian diffusion behavior (n < 0.5). The dipole-dipole interaction of the treatment molecules (positively charged) and nanoporous interface (negatively charged) (Figure 9) resulted in the physisorption or electrostatic force that have an influence on the characteristics of the vancomycin diffusion [47]. When the antibacterial drug moves from the deeper nanoporous structure with a weakened force that made drug transfer under the controlled gradient of the drug concentration, it is said to follow Fick’s law [48].

## 5. Conclusions

The titania nanotubes as a reservoir for the local drug delivery was successfully synthesized on the 3D-printed Ti-6Al-4V surface. The anodization time is found to have an influence on the TNTs morphology, length, and pore diameter. The FE-SEM analysis confirmed the formation of nanostructured TNTs with vertically oriented, closely packed, smooth and non-perforated walls. The synthesized TNTs enhanced the vancomycin release with highest cumulative release of 34.7% (69.5 ppm), higher than that of MICs at 24 h. The fabricated Ti-6Al-4V implant and the TNTs were found to have excellent wettability. The rough, nanostructured and nanoporous nature of the TiO_2_ formed on the Ti-6Al-4V surface is expected to facilitate the biocompatibility and osteointegration of the fabricated implant. Drug-loaded TNTs are expected to serve as an alternative to the current systemic drug delivery approach and the antibiotics used in treating infections.

## Figures and Tables

**Table 1:** Shows the measured inner pore diameters and length of the TNTs at 1, 2, 3 and 4 h anodization time. The measurements were obtained using Image J analysis and the FE-SEM micrographs. (Statical analysis with IBM Spss 22, One-way ANOVA).

Condition	Anodization Time (h)	Nanotube Size (nm)	
		Pore Diameter	Length
TNTs_1h	1	53 ± 15 *	1976 ± 56
TNTs_2h	2	108 ± 19 *	1938 ± 75
TNTs_3h	3	93 ± 20 *	2629 ± 145 *
TNTs_4h	4	114 ± 16 *	2492 ± 77 *

The nanotube size after anodization, * as a *p* value < 0.05 was considered statically significant.

**Table 2:** One-way ANOVA results of nanoporosities for the TNTs anodized at 1, 2, 3 and 4 h.

	Sum of Squares	df	Mean Square	F	*p*
Between Groups	453,951.984	3	151,317.328	488.539	0.000
Within Groups	246,548.565	796	309.734	-	-
Total	700,500.549	799	-	-	-

**Table 3:** One-way ANOVA results of TNT length anodized at 1, 2, 3 and 4 h.

	Sum of Squares	df	Mean Square	F	*p*
Between Groups	3,751,794.162	3	1,250,598.054	140.606	0.000
Within Groups	320,196.327	36	8894.342	-	-
Total	4,071,990.489	39	-	-	-

## Data Availability

The data presented in this study are available on request from the corresponding author.
